# Sulfotyrosine Recognition as Marker for Druggable Sites in the Extracellular Space

**DOI:** 10.3390/ijms12063740

**Published:** 2011-06-08

**Authors:** Joshua J. Ziarek, Maxime S. Heroux, Christopher T. Veldkamp, Francis C. Peterson, Brian F. Volkman

**Affiliations:** 1 Department of Biochemistry, Medical College of Wisconsin, 8701 Watertown Plank Road, Milwaukee, WI 53226, USA; E-Mails: jziarek@mcw.edu (J.J.Z.); mheroux@mcw.edu (M.S.H.); fpeterso@mcw.edu (F.C.P.); 2 Department of Chemistry, University of Wisconsin-Whitewater, 800 West Main Street, Whitewater, WI 53190, USA; E-Mail: veldkamc@uww.edu

**Keywords:** chemokine, sulfotyrosine, NMR, structure, alignment, drug discovery

## Abstract

Chemokine signaling is a well-known agent of autoimmune disease, HIV infection, and cancer. Drug discovery efforts for these signaling molecules have focused on developing inhibitors targeting their associated G protein-coupled receptors. Recently, we used a structure-based approach directed at the sulfotyrosine-binding pocket of the chemokine CXCL12, and thereby demonstrated that small molecule inhibitors acting upon the chemokine ligand form an alternative therapeutic avenue. Although the 50 members of the chemokine family share varying degrees of sequence homology (some as little as 20%), all members retain the canonical chemokine fold. Here we show that an equivalent sulfotyrosine-binding pocket appears to be conserved across the chemokine superfamily. We monitored sulfotyrosine binding to four representative chemokines by NMR. The results suggest that most chemokines harbor a sulfotyrosine recognition site analogous to the cleft on CXCL12 that binds sulfotyrosine 21 of the receptor CXCR4. Rational drug discovery efforts targeting these sites may be useful in the development of specific as well as broad-spectrum chemokine inhibitors.

## 1. Introduction

The normal function of chemokines is to direct the migration of cells during development, inflammation, and hematopoietic stem cell mobilization. As chemokines are secreted into the extracellular space, they bind to glycosaminoglycans present on the exterior of most cells, and establish a concentration gradient. Cells that express the G protein-coupled receptor (GPCR) specific for that chemokine migrate toward the origin of secretion. When the ability to traffic cells is hijacked, the chemokine network can maintain and coordinate many disease states. Chemokine signaling has been implicated in various autoimmune diseases, such as: multiple sclerosis, rheumatoid arthritis, and atherosclerosis (as reviewed by [1–3]). Though canonically associated with directing cancer metastases [4], the role of chemokines in tumor progression has been expanded to both growth and neovascularization (reviewed by [5]).

Chemokine signaling has been the target of drug discovery efforts almost since the initial identification of chemokines. These efforts have tried to identify small molecule therapeutics that target the seven transmembrane region of the receptors. However, the binding and activation of the chemokine receptor is a two-step process in which the *N*-terminus first binds the chemokine and then the chemokine activates the receptor by inserting its *N*-terminus into the transmembrane domain [6,7]. High affinity chemokine binding and recognition requires that the *N*-terminal region of the receptor be post-translationally modified by O-sulfation at specific tyrosine residues. Protein sulfation is catalyzed by two tyrosylprotein sulfotransferases (TPST-1 and -2) located in the trans-Golgi network [8]. All currently known chemokine receptors contain one or more tyrosines in the *N*-terminal region and the majority of these receptors are predicted to be sulfated using various algorithms. Although a precise recognition sequence has yet to be identified, the presence of one or more nearby acidic residues is strongly correlated with tyrosine sulfation by the TPSTs [9]. However, some prediction algorithms, like Sulfinator [10], do not predict sulfation at known sites, like sulfotyrosine (sTyr) 7 and 12 of CXCR4. Furthermore, because of the labile nature of this modification, sulfation sites have been experimentally confirmed for only five of the chemokine receptors: CXCR3, CXCR4, CCR2b, CCR5, and CX3CR1 [11–15]. Sequence alignment of chemokine receptors demonstrates the presence of a tyrosine residue approximately ten amino acids *N*-terminal to a conserved cysteine [9,16]; in most cases, this residue is predicted as an O-sulfation site.

**Figure 1:**
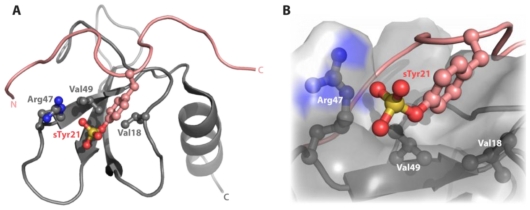
The CXCR4 sTyr21 binds to CXCL12 in a cleft formed by the N-loop and β3 strand. (**A**) The structure of CXCL12 (gray) in complex with the *N*-terminus of CXCR4 (salmon) illustrates that the architecture of the sulfotyrosine binding pocket requires both apolar and charged contacts (PDB 2K05). The side chains of CXCL12 residues Val18, Arg47, and Val49 all provided intermolecular NOEs to the CXCR4 sTyr21 residue-establishing the atoms are within 5 Å; (**B**) Surface representation underscores the presence of a binding cleft produced by the N-loop and β3 strand.

The molecular details of how chemokines recognize this conserved sulfotyrosine have only been determined for a sulfated CXCR4 *N*-terminal peptide bound to dimeric CXCL12 [17]. CXCR4 can be sulfated at positions 7, 12, and 21. While each forms specific contacts with CXCL12, tyrosine 21 corresponds to the conserved site of predicted sulfation. The structure of this complex revealed that the interface architecture involves both apolar and electrostatic contacts, which form a shallow cleft on the chemokine surface between the N-loop and β3 strand (Figure 1). In particular, the CXCL12 side chains of Val18, Arg47, and Val49 give rise to nuclear Overhauser effect (NOE) correlations with the ring of CXCR4 sTyr21 illustrating that the sulfotyrosine is within 5 Å of these residues. We recently demonstrated that small molecules binding the sTyr21 site of CXCL12 inhibit the functional interaction between chemokine and receptor *in vitro*. This demonstrates that, in addition to their receptors, chemokines may themselves be legitimate targets for drug discovery [18]. Because both the *N*-terminal tyrosine and the chemokine fold are highly conserved, we hypothesized that a sulfotyrosine-binding pocket analogous to the sTyr21 recognition site on CXCL12 may be present across the entire human chemokine superfamily.

To assess the likelihood that the chemokine family harbors a conserved sulfotyrosine binding site, we used a multiple sequence alignment to identify conserved residues in the vicinity of the sTyr21 pocket. Next, we inspected 3D chemokine structures and ModBase models to identify sidechains at other sequence positions that could substitute for differences in primary sequence. The results suggested that a putative sulfotyrosine-binding pocket was present in nearly all human chemokines. To test whether sulfotyrosine could be used as a probe to confirm the pocket, we monitored sulfotyrosine binding to CXCL12, XCL1, CX3CL1, and CCL5 by nuclear magnetic resonance (NMR) spectroscopy. Mapping of the residues most perturbed by sulfotyrosine onto the protein structure confirmed that a conserved recognitions site was present in representative members of each chemokine subfamily.

## 2. Methods

### 2.1. Chemokine Multiple-Sequence Alignment

Primary amino acid sequences of all human chemokines were submitted to the program ClustalW 2.1 to generate an alignment [19]. The consensus sequence was generated using the WebLogo server [20].

### 2.2. Chemokine Structures and Models

Previously determined chemokine structures were downloaded from the RCSB Protein Data Bank (www.pdb.org) [21]. Models of unsolved chemokines were obtained from, and are freely available at, ModBase (http://modbase.compbio.ucsf.edu/modbase-cgi/index.cgi) [22] and Modweb [23]. Only models with a MPQS score >1.1 were considered reliable for further analysis. The structures of all unsolved chemokines were reliably modeled with the exception of CCL25, CCL28, CXCL16, and CXCL17.

### 2.3. Structural Homology Modeling

Structures of each chemokine subfamily were subjected to pair-wise analysis with a representative chemokine (CCL5, CXCL12, or XCL1) from each subfamily. As modeled chemokines possess only a single solution, each NMR ensemble had to be reduced to one structure for alignment. First, an average structure was calculated using Molmol [24]; then, the RMSD between the mean structure and each member of the ensemble was calculated. The structure within the ensemble with the lowest heavy-atom RMSD was selected as the mean structure for that chemokine. Pair-wise alignment was performed using the DaliLite-pairwise option (Version 3.1) of the Dali Server [25].

### 2.4. Protein Expression and Purification

[U-^15^N]-CXCL12 expression and purification was carried out as previously described [26]. [U-^15^N]-XCL1cc3(1-93) was expressed and purified as previously described [27]. Expression and purification of [U-^15^N]-CCL5 and [U-^15^N]-CX3CL1 were conducted as described previously [28].

### 2.5. NMR Analysis

Each sample was prepared at previously published solution conditions to facilitate assignment transfer. Each sample contained 0.02% (v/v) NaN_3_ and 10% (v/v) D_2_O as the lock solvent. The CXCL12 sample contained 250 μM [U-^15^N]-CXCL12 in 25mM d-MES buffer (pH 6.8). [U-^15^N]-CCL5 was dissolved at 125 μM in 50 mM NaPO_4_ (pH 3.2). 250 μM [U-^15^N]-CX3CL1 was prepared unbuffered at pH 3.6. 250 μM [U-^15^N]-XCL1cc3 was prepared in 20 mM NaPO_4_ (pH 6.0).

Sulfotyrosine (H-Tyr(SO_3_H)-OH; Bachem) was titrated from 0–100 mM in 10 mM increments and monitored by 2D ^1^H-^15^N heteronuclear single-quantum coherence (HSQC) spectroscopy experiments. NMR analysis was performed on a Bruker 600 MHz spectrometer equipped with a TXI triple-resonance cryoprobe. Experiments with CXCL12 were collected at 37 °C and all other data were collected at 25 °C. Data was converted and processed using NMRPipe [29]. Previously published assignments for CXCL12 [26], CCL5 [30], XCL1 [27], and CX3CL1 [31] were transferred by visual inspection and chemical shift values were tracked using CARA [32]. Combined ^1^H-^15^N chemical shift perturbations were calculated as ((5Δδ_H_)^2^ + (Δδ_NH_)^2^)^0.5^, where δ_H_ and δ_NH_ are the amide proton and nitrogen chemical shifts, respectively.

## 3. Results and Discussion

### 3.1. Chemokine Primary Sequence Alignment

**Figure 2:**
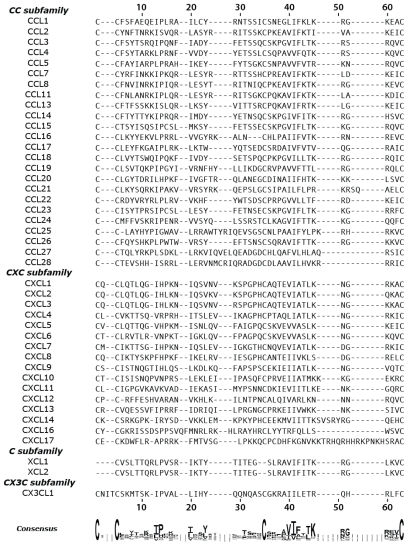
Primary sequence alignment of all human chemokines. Multiple sequence alignment was performed using ClustalW 2.1. The consensus sequence was generated from the WebLogo server [20].

Primary protein sequence alignment is a classic method of probing evolutionary conservation and homology. To identify regions of amino acid conservation within and between chemokine subfamilies, we performed a multiple-sequence alignment of all 43 human chemokines using ClustalW 2.1 (Figure 2) [19]. The *N*-terminus preceding the first conserved cysteine is highly disordered and the *C*-terminal helix orientation is dependent on solution condition and oligomeric state [33,34]; therefore, only the amino acids comprising the beta sheet of each chemokine were considered. The beta sheet residues were isolated by truncating the protein sequence prior to the first conserved cysteine and at the *C*-terminus following the conserved β3 cysteine. Amino acid positions will be hereafter referred to by their alignment position in Figure 2.

The consensus sequence reveals little overall homology aside from the conserved cysteine residues, which participate in structurally essential disulfide crosslinks. Conservation at a few other locations (e.g., proline at position 13, apolar residues at positions 23 and 40–42) may also preserve key structural features. However, conservation of apolar residues at the 13, 20 and 62 positions, may contribute to a common sulfotyrosine recognition site. The beta-branched hydrophobic residue at position 13 corresponds to Val18 in the CXCL12 N-loop, which contacts the phenyl ring of sTyr21 (Figure 1). The residue at position 20 in CXCL12, Val23, does not directly contact the CXCR4 sulfotyrosine but does contact Val49 a key residue in this cleft, which corresponds to sequence position 62. Further, all C, CC, CX3C, and 70% of CXC chemokines possess an apolar amino acid at position 62.

Examination of the alignment does not reveal strict conservation of polar contacts such as Arg47 from CXCL12. However, the presence of other positively charged residues in adjacent positions, between positions 45 and 59, suggests homologous electrostatic interactions. For example, 21 of 24 CC chemokines, 50% of CXC chemokines, and CX3CL1 maintain a basic residue at the position 60—analogous to CXCL12 Arg47.

### 3.2. Homology Modeling

Experimental structures have been determined for 28 of the 43 known human chemokines by NMR or X-ray crystallography. In order to compare the three-dimensional structural conservation across all chemokines, the structures of unsolved chemokines had to first be modeled. The servers Modweb and Modbase provide protein structure predictions based on a FASTA query sequence [23]. The reliability of all models is based on the MPQS value, which is a composite score comprised of sequence identity to the template, target coverage of the template, and three individual scores. The three scores E-value, Z-dope, and GA341 relate to the significance of the alignment between the target and the template, shape of the structure, and fold, respectively. Only models with a reliable MPQS score, >1.1, were used for subsequent analysis; if a chemokine had several reliable models, the version with the highest MPQS score was selected. This methodology provided 3D structures of all human chemokines except: CCL25, CCL28, CXCL16, and CXCL17 (Table 1). Examination of the template structure for each model reveals that chemokines of the same subfamily resulted in the highest scoring model except in the case of CCL18, CCL19, and CCL22 which all utilized a viral chemokine (PDB 1ZXT).

### 3.3. Structural Alignment

**Figure 3:**
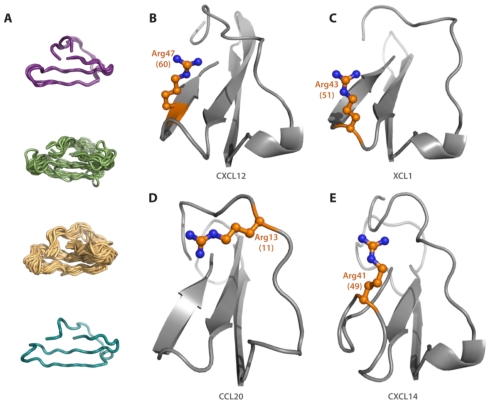
Chemokines maintain polar residues oriented toward the putative sulfotyrosine cleft. (**A**) Dali structural alignment for each chemokine subfamily, from top to bottom: C, CXC, CC, and CX3C chemokines. Polar residues oriented toward the putative binding pocket originate from four different positions; (**B**) 70% of the structures analyzed possess a basic residue at position 60, illustrated by CXCL12 Arg47; (**C**) Arginine 43 of the C chemokines, located at position 51, is oriented toward the cleft; (**D**) Several CC chemokines, CCL20 shown here, have basic residues in the N-loop positioned toward the binding cleft; (**E**) CXCL14 contains a G1 β-bulge in the β3 strand positioning Arg41 (position 49) toward the putative sulfotyrosine binding pocket.

Chemokines are known to adopt a canonical fold comprised of a three-stranded beta sheet followed by an alpha helix. Chemokines all activate G protein-coupled receptors and therefore may possess structural homology at the ligand:receptor interface. Previously determined chemokine structures and modeled chemokines were aligned using the Dali server. Pair-wise alignment was performed in each subfamily against a representative chemokine: CCL5, CXCL12, and XCL1. As CX3CL1 is the only CX3C family member, it was aligned against CXCL12. The quality of each alignment is assessed by a Dali Z-score, Cα RMSD, number of aligned residues, and percent sequence identity (Table 1) [35]. In general, each chemokine sequence was aligned over approximately 42 residues with 40 structurally equivalent residues. As the chemokine fold is highly conserved, it is not surprising that sequence identities between 14–55% still resulted in average backbone RMSD = 1.5–2.0 Å (Figure 3A). With the exception of CXCL11, all alignments resulted in a Z-score ≥ 2.0 indicating a significant degree of homology [36]. Overall, the lowest Z-scores can be correlated to poor sequence identity, such as the 9% identity between CXCL11 and CXCL12.

Visual inspection of the residues surrounding the putative pocket identified several potential polar contacts for sulfotyrosine coordination. As identified in the primary sequence alignment, 28 of the 39 human chemokine examined possess a basic residue at position 60; in all of corresponding structures this amino acid points toward the binding pocket for potential sulfate coordination (Figure 3B). The other 11 family members possess three varieties of structural features capable of rectifying this deficiency. Both XCL1 and XCL2 contain an Arg at position 51 that is directed toward the N-loop (Figure 3C). Five chemokines (CXCL2, CXCL9, CCL20, CCL21, and CCL24), which do not possess basic residues in the third beta strand, do maintain charged amino acids properly oriented in the N-loop (Figure 3D). CXCL11 and CXCL14 contain additional residues in β3 that pucker and allow basic residues at positions 52 and 49, respectively, to orient toward the putative pocket (Figure 3E). The turn between β2 and β3 of two CXC (CXCL10 and CXCL13) and three CC (CCL20, CCL21, and CCL24) chemokines contain several basic residues that are not optimally oriented in the current structures but could conceivably rearrange during receptor binding.

In addition to polar contacts, sulfotyrosine recognition also relies on hydrophobic interactions. The CXCR4 sTyr21 residue forms contacts with both CXCL12 Val18 and Val49 [17]. Primary sequence and structural alignment reveals strong conservation of apolar residues in the N-loop of all chemokines particularly at the position corresponding to CXCL12 Val18 (position 13). Furthermore, 35 of the 39 human chemokines possess a hydrophobic residue at position 62 (Figure 2) corresponding to CXCL12 Val49. Of the chemokines lacking this apolar amino acid in the β2 strand, CXCL9, CXCL10, and CXCL11 all maintain a non-polar residue (position 44) at the end of the β2 strand that could serve in its place; qualitatively, this apolar β2 residue is conserved throughout the CXC subfamily but exists primarily as a threonine in the rest of the chemokine family. Only CXCL14 is unique in that it does not contain corresponding residues to replace the hydrophobic β3 contact, but the functional target of this orphan chemokine remains unknown and may not rely on sulfotyrosine recognition. Overall, our results identify positively charged and apolar residues in the third beta strand and N-loop that are present in all human chemokine subfamilies suggesting a conserved sulfotyrosine binding pocket.

### 3.4. Sulfotyrosine Titration Identifies CXCL12 sTyr21 Binding Pocket

**Figure 4:**
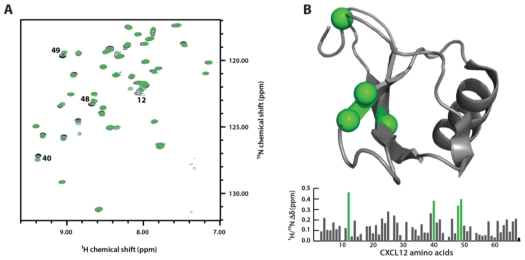
Sulfotyrosine can probe the CXCL12 sTyr21-binding pocket. (**A**) Overlay of HSQC spectra of CXCL12 in the presence of 0 mM (black), 10 mM (gray), and 30 mM (green) sulfotyrosine; (**B**) The change in the ^1^H-^15^N chemical shift was calculated and plotted as a function of CXCL12 residue number. Residues with the largest perturbations were mapped onto the CXCL12 structure and localize to the CXCR4 sTyr21-binding cleft.

The CXCR4 receptor is sulfated at three tyrosine residues that bind distinct locations of CXCL12. To test whether sulfotyrosine alone could act as a probe for the CXCL12 binding pockets, we monitored a titration using 2D ^1^H-^15^N HSQC spectroscopy. HSQC spectral overlays (Figure 4A) demonstrate significant chemical shift perturbations in the chemokine. The ^1^H-^15^N chemical shift perturbations were calculated and mapped onto the surface of CXCL12 previously solved at identical solution conditions in the absence of sulfotyrosine (PDB 2KEE; Figure 4B). The most significant chemical shift perturbations (purple) correspond to residues Arg12, Arg40, Gln48 and Val49, which border the CXCR4 sTyr21 binding pocket. These residues were previously identified by HSQC titration experiments in which the chemical shift difference between a sulfated and unsulfated CXCR4 peptide was used to identify the binding site [37]. The large chemical shift perturbations in residues His25 and Lys27 are consistent with the CXCR4 sTyr12 binding site. In the structure of dimeric CXCL12 bound to a triply-sulfated CXCR4 peptide, sTyr7 makes contacts with Val23 of the adjacent protomer suggesting a unique site on the CXCL12 monomer. At these experimental conditions CXCL12 exists in an equilibrium highly favoring the monomer, but sulfotyrosine addition did not produce any chemical shift perturbations suggestive of a monomeric CXCL12/sTyr7 binding pocket. Our results suggest that this assay is capable of identifying sulfotyrosine-binding pockets in other chemokines.

### 3.5. Sulfotyrosine Probe Highlights Similar Pocket in XCL1, CCL5 and CX3CL1 Chemokines

**Figure 5:**
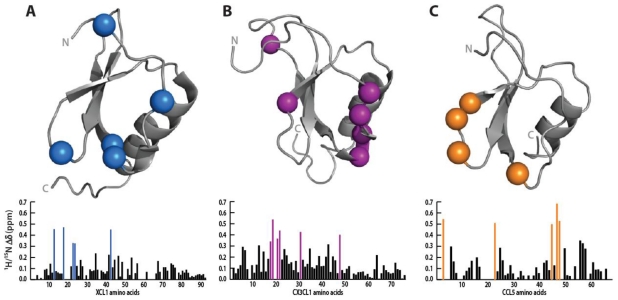
Sulfotyrosine localizes to the cleft between the N-loop and β3 strand. The difference in ^1^H-^15^N chemical shift in the absence and presence of 30mM sulfotyrosine were plotted as a function of residue number for XCL1, CX3CL1, and CCL5. In general, the residues with the largest perturbations (colored spheres) localize to the putative sulfotyrosine-binding cleft. (**A**) XCL1 had the largest chemical shifts in residues Ser13, Arg18, Arg43, and Val47; (**B**) CX3CL1 residues Lys18, Ile19, Val21, Ala22, Gln31, and Leu48 displayed the most significant chemical shift perturbations; (**C**) The chemical shifts in CCL5 also localized to the N-loop and β3 strand including residues Val3, His23, Lys45, Arg47, and Gln48.

Chemokine representatives of the C, CC, and CX3C chemokine were titrated with sulfotyrosine in 10 mM increments from 0 to 100 mM and monitored by 2D HSQC. The displayed chemical shift values were measured at 30 mM sulfotyrosine as higher concentrations resulted in pronounced non-specific perturbations as specific sites began to saturate. Similar to the titration of CXCL12, all three chemokines contained a subset of residues located primarily in the N-loop and β3 strand that displayed large chemical shift perturbations relative to the rest of the protein (Figure 5).

XCL1 is known to exist in a monomer-dimer equilibrium under physiological conditions, but only the monomeric form is capable of binding XCR1 [27]. Using a constitutively-locked monomer, XCL1cc3(1–93), we identified five residues (Ser13, Arg18, Arg23, Ile24, and Arg43) significantly perturbed by sulfotyrosine (Figure 5A). Our results support a sulfotyrosine binding pocket similar to CXCL12 in the cleft between the N-loop and β3 strand; however, there is currently no structural or mutagenic data mapping the XCR1 *N*-terminal binding site to confirm our results.

Sulfotyrosine titration produced significant perturbations in CX3CL1 residues Lys18, Ile19, Val21, Ala22, Gln31, and Leu48, which, with the exception of Gln31, cluster to the N-loop and β3 strand (Figure 5B). Titration of CX3CL1 with a CX3CR1 (1–19) peptide previously identified a role for Gln31 and Leu48 in *N*-terminus binding [31]. Mutagenesis experiments further illustrated a modest role for Lys18 in which a Glu substitution reduced affinity for CX3CL1 20-fold [38]. No mutagenesis experiments have specifically probed apolar residues in the putative binding cleft; however, our data suggests a critical role for these residues.

Although known to exist in a monomer-dimer equilibrium at low pH, the CCL5 spectrum contained only dimeric resonances consistent with the previously reported *K*_d_ = 35 μM [39]. Residues His23, Lys45, and Arg47 identified in our titration were previously shown to exhibit large chemical shift perturbations in the presence of a CCR5 *N*-terminal peptide sulfated at residues Tyr10 and Tyr14 (Figure 5C) [40].

## 4. Conclusions

The prevalence of both receptor tyrosine O-sulfation and the ubiquitous chemokine fold suggests the possibility of a conserved sulfotyrosine-binding site. In the extracellular *N*-terminus of most chemokine receptors, a tyrosine is present approximately ten residues away from a highly conserved cysteine [9,16] (e.g., Tyr21 of CXCR4). Using the CXCL12/CXCR4 complex as a guide, our structure-based homology analysis suggested that only the recognition site for sTyr21 is likely to be conserved in other chemokines. The sTyr21-binding cleft is located between the N-loop and β3 strand, which possesses both polar and hydrophobic contacts. This pocket, termed the chemokine groove, has been previously identified as a key mediator of receptor binding in CC, CX3C, and CXC subfamilies through mutagenesis and NMR binding experiments [31,41–47]. Overall, our analysis revealed few globally conserved sequence positions beyond the structurally essential cysteine residues located in the *N*-terminus and β3 strand that distinguish the four chemokine subfamilies [48]. However, several apolar and basic residues were found to cluster in and around the sulfotyrosine-binding pocket. Members from all four subfamilies, composing more than 70% of chemokines, possessed a basic residue at position 60. Further, 90% of all human chemokines possess an apolar residue at position 62.

Structural alignment not only confirmed the proper orientation and spatial position of residues identified by primary sequence alignment, but revealed compensatory amino acids in chemokines where the key sulfotyrosine recognition residues were not conserved. Absence of a basic residue at position 60 was remedied mainly through three alternative mutations. Most of the chemokines contained arginine or lysine residues at other positions within the β3 strand that position toward the pocket; several proteins also contained charged amino acids in the β2-β3 turn. The other deficient proteins, including CCL20, CCL21, and CCL24 all contain positively charged residues in the N-loop oriented toward the binding cleft. Indeed, CCL24 Arg15 experiences complete line broadening in an NMR titration with a CCR3 *N*-terminal peptide suggesting that this residue participates directly in receptor binding [49]. CCL25, CCL28, CXCL16, and CXCL17, which lack experimentally determined structures, could not be modeled due to low sequence homology with other family members. Interestingly, all four proteins still retain an apolar residue at position 62, and three of them (all except CXCL16) possess a basic residue at site 58 or 60 suggesting the presence of a cleft compatible with sulfotyrosine binding.

The problem of identifying the receptor sulfotyrosine binding pocket on chemokines has usually been solved by titrating sulfated peptides representing the *N*-terminal fragment of the receptor of interest into a chemokine sample and monitoring backbone amide chemical shift changes by HSQC NMR [37,40–42,49,50]. Several methods, such as solid-phase synthesis [50,51] and *in vitro* enzymatic sulfation [17,37,52], have been utilized to sulfate these peptides. However, these techniques are challenging due to low yields, the labile nature of the sulfate modification, and difficulties associated with separating complex mixtures of products. In an effort to produce a simpler probe, we titrated CXCL12, CCL5, CX3CL1, and XCL1 with free sulfotyrosine (H_2_N-Tyr(SO_3_)-CO_2_). These proteins were chosen as representatives of each subfamily because the binding site of each chemokine, with the exception of XCL1, had previously been probed with receptor peptides [31,37,38,40]. Each protein exhibited localized chemical shift perturbations in the chemokine groove correlating with previous peptide binding studies. In addition, although no XCL1/XCR1 binding information is published, the localization of perturbations to the analogous cleft suggests a similarly conserved sulfotyrosine-binding site. Interestingly, the strong perturbation in CCL5 Tyr3 suggests sulfotyrosine is a powerful probe for binding pocket identification regardless of whether the chemokine is in the native oligomeric state for receptor binding. Tyr3 is only located near the sulfotyrosine pocket when CCL5 is dimeric; however, only monomeric CCL5 interacts with the CCR5 *N*-terminus [40]. Although the sulfotyrosine probe binds too weakly (*K*_d_ ~10^−2^ M) for structural characterization, these results suggest that short sulfopeptides, which should be easier to produce, may possess sufficient specificity and affinity to enable structure determination by NMR [53].

Identification of specific sulfotyrosine binding pockets on chemokines could define a new category of targets for structure-based drug discovery. Currently, only a single chemokine/sulfopeptide structure, CXCL12/CXCR4, has been determined [17]. Using this structure we recently performed a high-throughput computational docking study to identify small molecules with the propensity to bind the sTyr21 site. The top ranking candidate ligands were then tested for binding by NMR, and one compound with a 64 μM affinity was found to inhibit CXCR4-mediated calcium flux signaling of THP-1 monocytes [18]. Similar docking studies against the XCL1 site defined by sulfotyrosine chemical shift mapping have identified small molecule ligands that bind the target site and are currently being tested for inhibition of XCL1/XCR1 interactions [54].

Overall, our results show that the chemokine superfamily possesses a conserved sulfotyrosine binding site, critical for high-affinity interactions that can potentially be targeted for the design of specific or broad-spectrum inhibitors. Since the discovery of the first chemokine, CXCL8, almost 25 years ago a total of 43 human chemokines have been identified. As only three new chemokines have been isolated in the last decade [48], it is generally believed that most human chemokines have been discovered. Thus, we conclude that the chemokines lacking a potential sulfotyrosine recognition site represent exceptions to the general rule. The chemokines and their receptors are broadly expressed and relatively promiscuous with two or more partners in most cases. As a consequence, inhibitors targeting a specific receptor may not have the optimal specificity, since they may interfere with signaling of multiple chemokine ligands, or a second receptor could coordinate chemotaxis toward a given site of chemokine secretion. If the chemokine ligands can instead be inhibited by blocking the sulfotyrosine-mediated receptor interaction, novel inhibitors might be designed with favorable therapeutic properties. In addition, high-affinity ligands could also be adapted for use as diagnostic molecules for imaging of chemokine levels in either research or clinical settings. Although the structural similarities outlined in this article and elsewhere suggest that sulfotyrosine-directed selective blocking of individual chemokines could be challenging, the potential for identifying broad-spectrum inhibitors represents a powerful complementary strategy.

## Figures and Tables

**Table 1:** A list of all human chemokine structures used. The Protein DataBank (PDB) accession number for all chemokines is listed in Table 1(a). Chemokines in Table 1(b) were modeled from the indicated template PDB structures and are available from the ModBase Database using the Accession ID. The MPQS score is a composite score to describe the model quality, generally scores ≥1.1 are reliable. Statistics from pair-wise alignment for each chemokine against a representative from its subfamily (CCL5, CXCL12, and XCL1) are summarized by the Dali Z-score (values ≥2.0 are reliable), Cα RMSD, and percent sequence identity. CX3CL1 was aligned against CXCL12.

(a)				
Chemokine	PDB ID	Dali Z-score	Cα RMSD (Å)	Sequence Identity (%)
XCL Subfamily	2HDM			
XCL 1				
CCL Subfamily				
CCL1	IEL0	3.6	2.3	30
CCL2	1DOM	5.6	1.3	25
CCL3	QB53	5.3	1.6	50
CCL4	1HUM	5.4	1.7	55
CCL5	1HRJ			
CCL7	1BO0	5.1	1.5	28
CCL8	1ESR	5.8	1.5	33
CCL11	1EOT	5.1	1.5	30
CCL13	2RA4	5.8	1.5	28
CCL14	2Q8T	6.1	1.3	40
CCL15	2HCC	5.3	1.5	50
CCL17	1NR4	5.6	1.5	38
CCL20	2JYO	5.7	1.4	30
CCL23	1G91	5.3	1.5	40
CCL24	1EIG	5.1	1.6	28
CCL26	1G2S	5.8	1.5	43
CCL27	2KUM	4.0	2.0	21
CXCL subfamily				
CXCL1	1MSG	2.1	2.8	14
CXCL2	1QNK	5.2	2.0	20
CXCL4	1F9A	4.8	1.9	26
CXCL7	1NAP	5.2	1.8	23
CXCL8	1ILQ	4.0	2.0	21
CXCL10	1LV9	3.7	2.6	15
CXCL11	1RJT	1.7	2.6	9
CXCL12	2KEE			
CXCL14	2HDL	4.6	2.6	20
CX3CL subfamily				
CX3CL1	1B2T	4.0	2.3	18

(b)						
Chemokine	Mod Base ID	Template PDB	MPQS Score	Dali Z-score	Cα RMSD (Å)	Sequence Identity (%)
XCL subfamily						
XCL2	Q9UBD3	1J8I (XCL1)	1.85	5.1	1.2	97
CCL subfamily						
CCL16	O15467	2Q8R (CCL4)	1.16	5.6	1.1	41
CCL18	P55774	1ZXT (viral)	1.40	5.6	1.1	35
CCL19	Q99731	1ZXT (viral)	1.21	5.4	1.5	33
CCL21	O00585	1ESR (CCL8)	1.11	4.8	1.5	33
CCL22	O00626	1ZXT (viral)	1.25	5.6	1.5	33
CXCL subfamily						
CXCL3	P19876	1QNK (CXCL2)	1.70	4.7	2.0	18
CXCL5	P42830	1TVX (CXCL7)	1.27	5.0	1.9	23
CXCL6	P27784	1TVX (CXCL7)	1.22	4.4	1.8	26
CXCL9	Q07325	1GNK(CXCL2)	1.18	4.8	1.8	28
CXCL13	Q43927	3IL8(CXCL8)	1.26	4.3	1.9	26
